# Valerianate of Zinc a Powerful Antispasmodic

**Published:** 1844-12-28

**Authors:** 


VALERIANATE OF ZINC A POWERFUL ANTISPASMODIC.

BY M. DEVAY.

The valerianate of zinc has lately been highly commended in several Italian Journals as a valuable therapeutic agent in the cure of neuralgic affections. M. Devay having made numerous trials of its powers in these affections, arrived at the conclusion, that it was a remedy of no mean powers, which deserved to be more generally used. It appears, however, from the cases which he records, that its curative powers are confined to those in which the disease depends on a purely nervous affection, and in the excitable nervous affections which accompany chlorosis, in facial neuralgia, or tic-douloureux, not depending on an organic cause, in hemicrania, and even in some cases of satyriasis and epilepsy, its therapeutic virtues have been powerfully manifested. Several cases are recorded by M. Devay of its curative powers. It seems to act as a powerful antispasmodic, combining the powers of valerian and of zinc. The dose is half a grain two to four times daily.

The valerianate is prepared in the following manner. The fresh roots of valerian are distilled, when the valerianic acid comes over along with the essential oil. This oil is separated, and the distilled water has its acid saturated by carbonate of potash. Solution of caustic potash is also agitated with the essential oil, and both fluids are mixed together. The valerianate of potash not being volatile, allows the most of the water to be driven off, as well as that portion of the volatile oil which has not united with the alkali. When the valerianate of potash is sufficiently concentrated, it is introduced into a small retort, and a sufficient quantity of dilute sulphuric added to unite with the potash. Heat is then carefully applied, and the volatile valerianic acid distils over in a pure state, partly dissolved in a small quantity of water, partly as an oily hydrate. It is then mixed with carbonate of zinc, and the union aided by heat. It is then filtered, and as the fluid cools the crystals of the valerianate of zinc are deposited. The mother liquor is to be evaporated till all the salt is obtained. —*Ibid, from Gazette Médicale de Paris.*

